# Supplementary material for: Allele-specific immune gene quantification and expression analysis in single-cell RNA-seq data
Source: NAR Genom Bioinform. 2025 Nov 11;7(4):lqaf149. doi: 10.1093/nargab/lqaf149 (PMC12604667; doi:10.1093/nargab/lqaf149)
Supplement: lqaf149_Supplemental_Files [file lqaf149_supplemental_files.zip › supplement_alajami.pdf]

Supplementary data

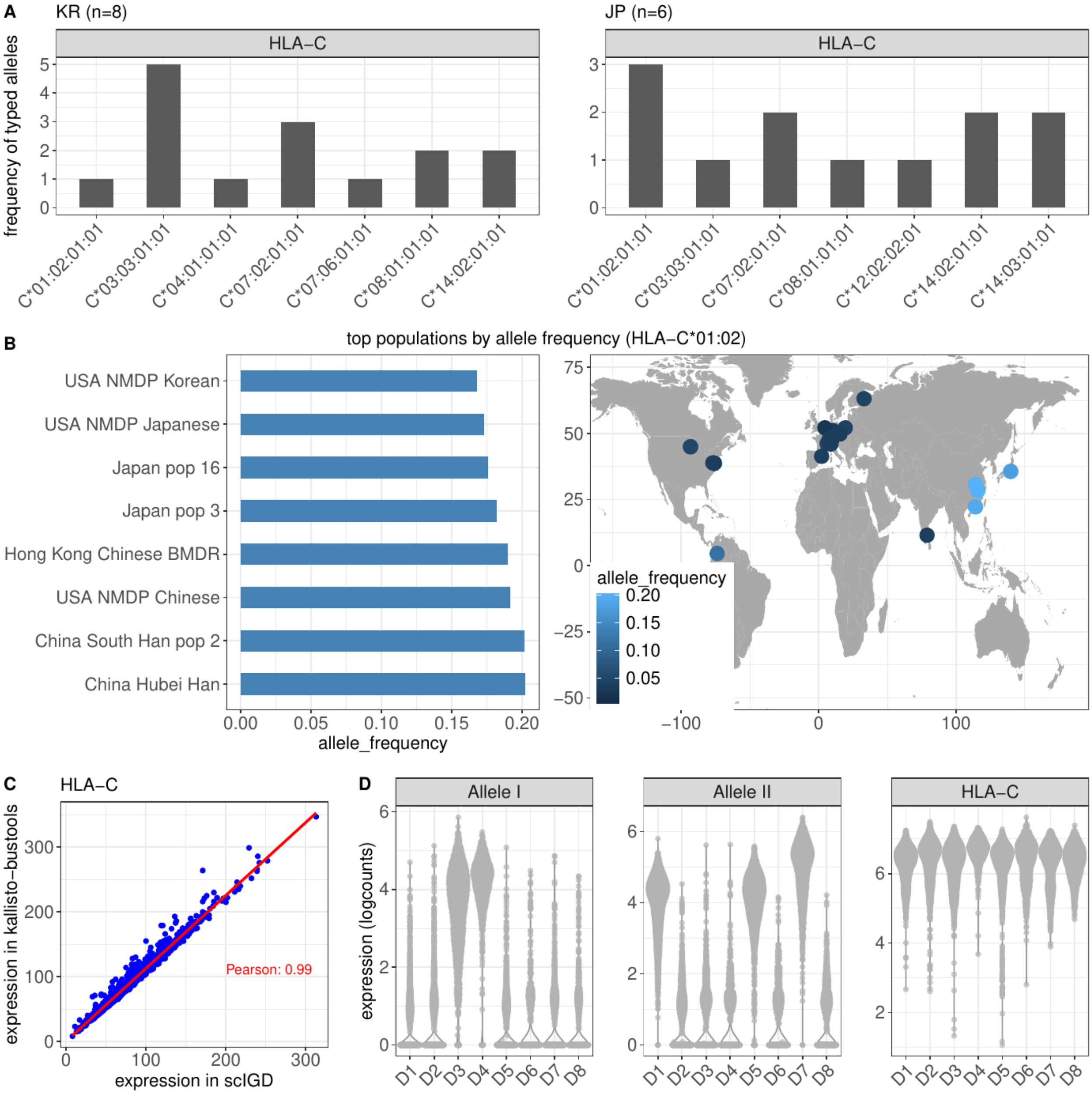

Supplementary Fig. 1. Validation of HLA allele-typing, specificity, and quantification in amplicon-based data
A) Absolute frequency of *HLA-C* alleles typed in a subset of the AIDA dataset [19], including 14 samples: 8 Korean (KR - left panel) and 6 Japanese (JP - right panel) individuals. HLA allele-typing was performed using the scIGD workflow, with no prior information about ethnicity or population. **B)** Relative frequency of the *HLA-C*01:02* allele group identified in (A) in different human reference populations from the Allele Frequency Net Database (AFND) [27]. The bar graph on the left shows the 8 highest relative frequencies in AFND. The map on the right shows a visualization of the allele group frequencies in human populations worldwide. **C)** Comparison of *HLA-C* gene expression quantification using scIGD (x-axis) versus kallisto-bustools (y-axis). Raw gene expression counts in the D1 sample of the Multiple Myeloma dataset are shown. HLA gene expression in scIGD is calculated by aggregating the counts of alleles corresponding to each gene. Pearson correlation coefficient is indicated. **D)** Assessment of HLA allele specificity in the Multiple Myeloma dataset, containing 8 donors. The plot displays normalized expressions of two different *HLA-C* alleles and the *HLA-C* gene across all donors. Alleles identified in specific donors exhibited notably higher expression levels compared to those in other donors. In contrast, the expression of the *HLA-C* gene remained uniform across all donors. Note: Only two alleles are shown here for illustrative purposes, but additional alleles were also typed from these 8 donors.

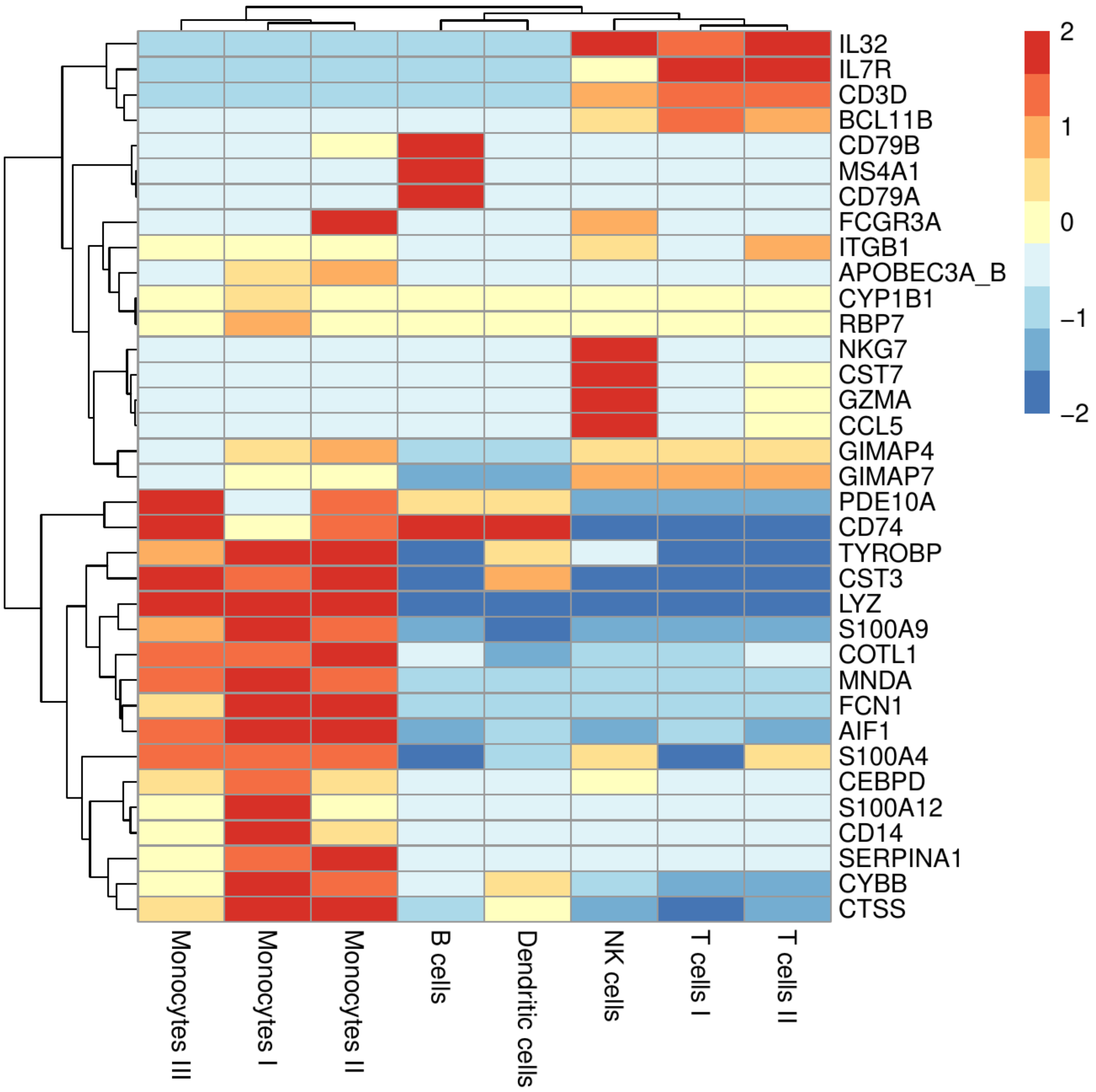

Supplementary Fig. 2. Gene expression profiling for cluster annotation in 20k PBMC dataset
Heatmap showing the centered mean expression values of the most important marker genes for the different single-cell clusters in the 20k PBMC dataset. Cluster annotation was achieved using these marker genes.
